# On the Inside

**DOI:** 10.1093/plphys/kiab316

**Published:** 2021-09-04

**Authors:** Peter V. Minorsky

**Affiliations:** School of Health and Natural Sciences, Mercy College, Dobbs Ferry, New York, USA

## UV irradiation induces a stocky phenotype in absence of stress

Global climate change and the recovery of the stratospheric ozone layer are resulting in novel combinations of temperature, water availability, and solar ultraviolet (UV) radiation. Although much research has concerned the stressful effects of UV irradiation on plants, there is also a heightened appreciation of the effects of UV irradiation on normal growth processes. Moreover, there is now a better understanding of the mechanism underlying the sensing of UV-B and UV-A wavelengths by a variety of plant photoreceptors, including the phototropins, cryptochromes, and UV RESISTANCE LOCUS 8 (UVR8). However, our understanding of the downstream regulatory interactions that modify UV-A and UV-B responses is only slowly emerging. UV light has been shown to induce a stocky phenotype in many plant species but is it a stress response? Qian et al. (pp. 378–395) have investigated this effect in regard to specific UV wavebands (UV-A or UV-B) and the cause for this dwarfing, which they show occurs under conditions that do not induce any of the typical biochemical indications of UV stress. UV-A- or UV-B-enrichment of growth light both resulted in a smaller cucumber (*Cucumis sativus*) phenotype, exhibiting decreased stem and petiole lengths and leaf area. The effects were greater in plants grown in UV-B- than in UV-A-enriched light. In plants grown in UV-A-enriched light, decreases in stem and petiole lengths were similar irrespective of tissue age. In contrast, in the presence of UV-B radiation, stems and petioles were progressively shorter the younger the tissue. Also, plants grown under UV-A-enriched light significantly reallocated photosynthates from shoot to root and had thicker leaves with decreased specific leaf area. Thus, UV-induced dwarfing, which displayed different phenotypes depending on UV wavelengths, occurred in healthy cucumber plants, implying a regulatory adjustment as part of the UV acclimation processes involving UV-A and/or UV-B photoreceptors.

## Trp-derived defenses against a mite

The plant hormone jasmonate (JA) accumulates in herbivore-damaged plants and triggers a variety of defense responses in different plant species, which may include the synthesis of various defensive metabolites, volatiles, and/or proteins. In the Brassicaceae, including Arabidopsis (*Arabidopsis thaliana*), glucosinolates are the main defense compounds against herbivores. Aliphatic and indole glucosinolates, which are derived respectively from the amino acids Met and Trp, are the most abundant glucosinolates in Arabidopsis. These compounds have varying defensive specificities: some herbivores are sensitive to aliphatic glucosinolates, some to indole glucosinolates, and some to both classes of compounds. Although Trp-derived metabolites are known to limit Arabidopsis infestation by the two-spotted spider mite, *Tetranychus urticae*, the downstream phytochemicals responsible for Arabidopsis protection against *T. urticae* are unknown. Widemann et al. (pp. 116–132) have used Arabidopsis mutants disrupted in the synthesis of Trp-derived secondary metabolites to identify phytochemicals involved in the defense against *T. urticae*. Their findings reveal that all three of the major forms of indole glucosinolates in Arabidopsis can limit *T. urticae* herbivory, but they must be processed by myrosinases in order to hinder *T. urticae* oviposition. Finally, the authors demonstrate that besides indole glucosinolates, there are additional JA-regulated defenses that control *T. urticae* herbivory. Together, these results reveal the complexity of Arabidopsis defenses against *T. urticae* that rely on multiple indole glucosinolates, specific myrosinases, and additional JA-dependent defenses.

## Insights into leaf margin formation

The wondrous variations of leaf forms seen in nature are in no small part due to differences in the shapes of their margins, which can be categorized as smooth, serrated, or lobed. During shoot development, leaves begin as peg-like primordia on the flanks of the shoot apical meristem, while leaflets are initiated from the marginal region of the primordia and later form their final shape. Auxin plays a major role in many aspects of leaf development, including margin formation. The auxin transport carrier PIN-FORMED1 (PIN1), for example, has a polar localization and creates auxin activity maxima, which are involved in the development of serrations along the leaf margin. Pharmacological or genetic perturbations of polar auxin transport or activity results in the failure to form leaf serrations and a plant that exhibits toothless leaves. The ectopic expression of these genes or exogenous auxin application results in more serrations or generates extra leaflets. In simple-leafed species such as cotton (*Gossypium hirsutum*) and rapeseed (*Brassica napus*), the transcription factor LATE MERISTEM IDENTITY1 (LMI1) and its putative paralogs are responsible for determining the formation of leaf lobes. Wang et al. (pp. 218–235) now turn their attention to the possible role of LMI1 in the formation of leaf margins in barrelclover (*Medicago truncatula*), a compound-leafed, model legume. Loss-of-function mutations of the putative paralogs *MtLMI1a* and *MtLMI1b* are essentially co-expressed along the leaf margin and redundantly promote the development of leaf margin serrations, as revealed by the relatively smooth leaf margin in their double mutants. MtLMI1s directly activate expression of SMOOTH LEAF MARGIN1 (SLM1), which encodes an auxin efflux carrier, thereby regulating auxin distribution along the leaf margin. The interactions of MtLMI1s with other developmental proteins are discussed.

## Chloroplast ribosomes and tobacco vein banding mosaic virus infection

Potyviruses (genus Potyvirus, family Potyviridae) form the largest plant RNA virus group and cause substantial economic losses to crop production worldwide. The potyvirus genome is a positive-sense single-stranded RNA that encodes 11 proteins. Chloroplast proteins are often recruited by plant viruses to support viral replication and movement. However, the mechanism by which chloroplast proteins regulate potyvirus infection remains largely unknown. Tobacco vein banding mosaic virus (TVBMV) infection has previously been shown to cause differential expression of many chloroplast-related genes in *Nicotiana benthamiana.* Cheng et al. (pp. 174–186) now report that *N. benthamiana* ribosomal protein large subunit 1 (NbRPL1), a chloroplast ribosomal protein, localized to the chloroplasts via its N-terminal 61 amino acids (transit peptide), interacts with tobacco vein banding mosaic virus (TVBMV) nuclear inclusion protein b (NIb), an RNA-dependent RNA polymerase. Upon TVBMV infection, NbRPL1 is recruited into the viral replication complexes in chloroplasts. Silencing of *NbRPL1* expression reduced TVBMV replication. NbRPL1 competes with the autophagy-related protein, NbBeclin1 to bind NIb, and reduces the NbBeclin1-mediated degradation of NIb. These results suggest that NbRPL1 interacts with NIb in the chloroplasts, reduces NbBeclin1-mediated NIb degradation, thereby enhancing TVBMV infection

## Vegetative phase change in pine

Many perennial woody plants experience a prolonged juvenile phase during which they cannot reproduce sexually. In Norway spruce (*Picea abies*), for example, about 20–25 years are required to reach reproductive age, while *Pinus tabuliformis* needs 5–7 years to complete its juvenile phase. Despite considerable progress in the understanding of flowering time control in model plants such as *Arabidopsis thaliana*, the mechanism underlying the developmental passage from juvenility to reproductive maturity in perennial trees, especially conifers, remains largely unclear. Previously, it has been proposed that the *DEFICIENS-AGAMOUS-LIKE 1* (*DAL1*) gene might be a possible mediator of phase change in Norway spruce, as its expression is stimulated in the mid-juvenile phase and increases with age. To gain further insight into the molecular basis of the juvenile phase change in conifers, Chen et al. (pp. 247–262) examined the transcriptome of *P.* *tabuliformis* at seven different ages. Their results reveal that the phase change from juvenility in *P. tabuliformis* occurred unexpectedly rapidly rather than as a slow, gradual progression. Expression analysis in *P. tabuliformis* and a late-cone-setting *P. bungeana* mutant showed a tight association between *PtMADS11*, a gene that encodes a MADS-box transcription factor, and reproductive competence. The authors present evidence that *MADS11* and *DAL1* physically interact to coordinate the phase change from juvenility in conifers. Other factors implicated in the juvenile-to-adult transition in Arabidopsis were also considered. miR156 and miR172, which correlate strongly with age in perennial broadleaf trees, were also studied, as was another MADS-box protein called SOC1 that is involved in regulating the juvenile-to-adult transition in Arabidopsis. Overexpression of *PtMADS11* and *PtDAL1* partially rescued the flowering of 35S::miR156A and *spl1,2,3,4,5,6* mutants in Arabidopsis, but other findings suggest that PtMADS11 and PtDAL1 play different roles in flowering regulatory networks in Arabidopsis. *PtMADS11* could not alter the flowering phenotype of *soc1-1-2*, indicating it may function differently from AtSOC1 in Arabidopsis. Thus, the *MADS11* gene in pine is a regulatory mediator of the juvenile-to-adult transition with functions differentiated from the angiosperm SOC1.

## Moss gametophore development

Since the shoot-like gametophores of mosses and shoots of flowering plant have evolved independently, the underlying genetic network governing their development is distinct. However, both types of structures exhibit similar developmental responses to hormones such as auxin. Mohanasundaram et al. (pp. 203–217) describe a Tnt1 insertional mutant (*short-leaf; shlf*) in the model moss *Physcomitrium patens* that demonstrates the involvement of a novel bryophyte-specific gene in apical dominance, leaf development, plasmodesmatal (PD) frequency, and auxin distribution pattern in the moss gametophore. Based on a comprehensive phenotypic analysis, differential auxin accumulation and response at the gametophore apex, and PD frequency determinations, the authors show that the reduced apical dominance in *shlf* gametophores is associated with impaired auxin distribution. Whole-genome sequencing and functional characterization of candidate genes revealed that a unique bryophyte-specific gene, *SHLF*, is responsible for the *shlf* (short-leaf) phenotype. Phylogenetic analysis established that SHLF represents a novel family of tandem direct repeat-containing proteins that are conserved only among mosses and liverworts. In summary, the novel bryophyte-specific gene SHLF regulates apical dominance, leaf length, PD frequency, auxin distribution pattern, and governs overall gametophore development in moss.

